# Markerless gait analysis for children and adolescents with juvenile idiopathic arthritis using a machine learning pipeline

**DOI:** 10.1186/s12969-026-01211-w

**Published:** 2026-04-28

**Authors:** Aseem Behl, Dominik Papies, Winfried Ilg, Mareen Kraft, Andrea Bevot, Sandra Hansmann

**Affiliations:** 1 Faculty of Economics and Social Sciences, University of Tübingen, Nauklerstr. 47, 72074 Tübingen, Germany; 2 Machine Learning Cluster of Excellence, University of Tübingen, Tübingen, Germany; 3 Section Computational Sensomotorics, Hertie Institute for Clinical Brain Research, Otfried-Müller-Str. 27, 72076 Tübingen, Germany; 4 Centre for Integrative Neuroscience (CIN), University of Tübingen, Otfried-Müller-Str. 25, 72076 Tübingen, Germany; 5 Clinic for Paediatrics and Adolescent Medicine, Department of Neuropaediatrics, General Paediatrics, Diabetology, Endocrinology, Social Paediatrics, University Hospital Tübingen, Hoppe-Seyler-Str. 1, 72076 Tübingen, Germany

**Keywords:** Juvenile Idiopathic Arthritis, Gait analysis, Deep learning, Markerless motion capture, Clinical monitoring

## Abstract

**Background:**

Musculoskeletal diseases, such as Juvenile Idiopathic Arthritis (JIA), can lead to altered joint mobility and changes in gait pattern. While marker-based motion capture systems are considered the gold standard, their use is limited by high costs, technical complexity, time-intensive procedures, and specialized staff requirements. This study proposes and evaluates the feasibility of a markerless gait analysis approach for children of all ages, based on a machine learning pipeline, validates it against marker-based systems, and describes JIA-related gait differences.

**Methods:**

Sagittal-plane gait videos were recorded using a standardized setup. A Mask R-CNN-based detector generated a bounding box around the body in each video frame. Within this box, a TCFormer model identified positions of predefined anatomical landmarks. Joint angles were computed from these landmarks on a frame-by-frame basis and assembled into continuous gait cycle trajectories. For validation, a subset of 12 participants was recorded simultaneously using a marker-based motion capture system and conventional video-recording. To ensure direct comparability between the two methods, physical markers in video-recordings were automatically detected and removed using a deep learning-based object detection and inpainting approach. Statistical comparisons were performed using Statistical Parametric Mapping. The estimated kinematic data were compared between JIA patients with lower-extremity involvement and typically developing (TD) peers, stratified by age and disease activity.

**Results:**

Videos from 108 participants (60 JIA; 48 TD; age 1.8–17.4 years) were included. There was robust agreement between joint movement patterns of the markerless and the marker-based approach. Across all age groups, JIA patients with active arthritis had a reduced knee range of motion compared to TD (46.3$^{\circ}$ vs. 49.8$^{\circ}$). Overall, differences in joint angle trajectories between groups remained limited.

**Conclusions:**

The proposed single-camera, markerless pipeline can provide reliable and valid gait data using standard video equipment in children. Differences in gait patterns between JIA patients and TD peers were generally small, likely due to effective disease control. This method is adaptable across all ages, may facilitate enhanced gait monitoring, increase knowledge of functional abnormalities in JIA, and support therapeutic decisions. In the long run, it may also reduce costs by lowering infrastructure and setup requirements.

## Background

Independent gait is a key developmental milestone, typically achieved by children at approximately 12–18 months of age [1]. During the first 6 months of independent walking, gait is more variable, slower, less stable, and characterized by a wider base of support [2]. Around the age of 10 years at the earliest, the characteristics of adult walking are achieved and gait velocity matures [3]. School children on average walk up to 16,000 steps a day [4], and walking is a critically important part of everyday life. If walking is accompanied by pain or unsteadiness due to disabling diseases, this might have a strong detrimental impact on the emotional and physical well-being of children and their development [5, 6]. It is therefore important to be able to easily and reliably diagnose possible impairments to a child’s walking ability at any age and stage of development.

Neurodevelopmental and musculoskeletal disorders, such as juvenile idiopathic arthritis (JIA), significantly impair childhood movement patterns, mobility, physical activity, and development [7, 8]. As the most prevalent pediatric rheumatic condition, JIA, a chronic inflammatory joint disease, encompasses seven subgroups according to the International League of Associations for Rheumatology (ILAR) with onset before the age of 16 [9–11]. Although any joint can be affected, inflammation frequently involves weight-bearing joints [9, 12]. Clinical assessment of JIA prioritizes disease activity through physician and patient/ parent global assessments, active joint counts, passive range of motion (ROM) evaluation, and functional ability metrics [13–15]. While these tools effectively monitor therapeutic response and guide management, they may overlook subtle biomechanical adaptations. Arthritis-induced joint dysfunction frequently alters kinetic chain loading, impacting both affected and adjacent joints [16]. Such compensatory mechanisms manifest as deviations in gait parameters, including velocity, trunk inclination, and postural stability, potentially contributing to physical limitations and early fatigue in daily activities [17, 18].

Pain and kinesiophobia in children and adolescents with JIA can impair physical performance, gait function, and well-being, and may lead to persistent gait adaptations [19–22]. Mild to moderate walking disability persists in approximately one quarter of patients five years after disease onset [23]. While early-onset JIA primarily impairs motor development and functional abilities in young children [24, 25], affected adolescents present with higher rates of fatigue, depression, anxiety, and severe physical disabilities [26, 27]. Regardless of age, more severe physical disability and greater functional limitations increase the risk of reduced health-related quality of life and participation [28].

To date, only a few studies have systematically investigated objective gait changes in children with JIA utilizing a variety of methods. The methods include clinical tests such as the six-minute walk test to assess walking speed and performance parameters, pressure sensors and accelerometers to evaluate strides, symmetry and velocity, and three-dimensional instrumented gait analysis [29–33]. The main findings were a reduction in walking speed and step length, increased flexion in the knee and hip joints, and a reduced ROM in the ankle joints resulting in a flexed movement pattern, with gait changes described as a persistent symptom during both the active phase of the disease and beyond [17]. Therefore, gait analysis has the potential of being an additional powerful tool in diagnostic and therapeutic management of JIA. Marker-based three-dimensional (3D) instrumented gait analysis, currently the gold standard, can be used to precisely analyze and characterize various aspects of gait, including spatiotemporal parameters (e.g., stride length and frequency), kinematic data (e.g., three-dimensional joint angles and ROM), and kinetic variables (e.g., ground reaction forces and joint moments) [34]. Measurements are performed using an optoelectronic stereophotogrammetric system consisting of several infrared cameras that can detect and track reflective markers [35].

Importantly, marker-based motion capture remains impractical for routine clinical use due to high costs, laboratory requirements, and the need for specially trained staff to perform labor-intensive marker placement [36]. Especially, in pediatric populations, the physical presence of markers may alter natural movement patterns and potentially bias the results, while the prolonged, uncomfortable instrumentation process often leads to poor patient compliance [37, 38].

Recent advances in deep-learning-based pose-estimation have markedly improved the automatic recognition and tracking of human motion in ordinary video recordings [39–41]. Deep neural networks in particular are well suited to identify anatomical landmarks and gait events, enabling automated estimation of joint angles and gait parameters [42]. Video-based gait analysis provides a quantifiable and accessible alternative, with initial evidence demonstrating good agreement with marker-based 3D systems regarding spatiotemporal parameters as well as kinematic parameters [43, 44]. Building on these developments, this study evaluates whether single-camera videos can provide clinically useful kinematic gait measures across pediatric ages.

The aims of the present work are to (1) propose a single-camera, video-based approach designed to provide clinicians with a framework for gait analysis across all age groups that requires minimal technical equipment and setup time and is nevertheless accurate, (2) validate this approach against marker-based gait analysis in typically developing (TD) children and patients with JIA, and (3) to identify gait differences by age and disease activity.

## Material and methods

**Fig. 1:**
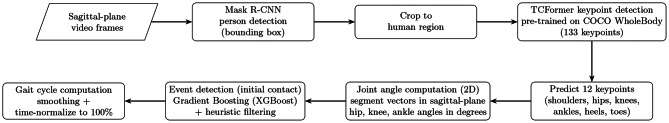
Workflow of the proposed video-based gait analysis pipeline. A pre-trained Mask R-CNN detects the person and provides a bounding box per frame for cropping. TCFormer keypoints are predicted on the cropped frames; only 12 anatomically relevant keypoints are retained for subsequent 2D joint-angle computation in the sagittal image plane. Initial contact events are detected using a Gradient Boosting classifier on joint-angle features with post-processing to remove duplicate detections. Gait cycles are segmented, smoothed, time-normalized to 100%

A single-center cohort study was conducted with a consecutive group of children and adolescents with JIA and TD. The methodological process involved four main steps. First, raw data were collected using a standardized video recording setup for a total of 108 participants. During the second step, machine learning approaches were used to recognize key-point locations of important anatomical landmarks and the initial contact event to define the individual gait cycles. Both steps were combined to calculate continuous hip, knee, and ankle joint angles during gait cycles. Third, the validity of the proposed approach was evaluated against the gold-standard optoelectronic system. Finally, the kinematic data generated from the validated framework were used to identify and describe gait deviations across the cohort. The proposed video-based gait analysis pipeline is outlined in Fig. 1 and described in detail in the subsequent sections.

### Participants

The JIA cohort was recruited during their clinical care at the University Hospital Tuebingen. Inclusion criteria for the JIA cohort were a diagnosis of JIA according to the ILAR classification [11] with lower limb involvement. Clinical data were obtained from the institutional electronic documentation system. The physician global assessment of disease activity (Physician GA; 0–10 Visual Analog Scale [VAS], 0 = no activity, 10 = maximum activity) and the patient/ parent global assessment of well-being (Patient/ Parent GA; 0–10 VAS, 0 = best, 10 = worst) were collected during routine care following [14]. Pain was assessed by patients or parents using a Numerical Rating Scale (NRS; 0 = no pain, 10 = maximum pain) as part of the German version of the Childhood Health Assessment Questionnaire (CHAQ) [45]. Physical and musculoskeletal examinations were performed by an independent pediatric rheumatologist who was not involved in this study.

The control group consisted of TD siblings or friends of patients treated at the hospital. None of them had neurological or musculoskeletal disorders, and no participant in either group reported injuries or conditions that might impair walking. In both groups, individuals who relied on walking aids or were unable to walk 50 meters without support were excluded.

The study was conducted in accordance with the Declaration of Helsinki and approved by the Ethics Committee at the Medical Faculty and the University Hospital Tuebingen (project No. 001/2020BO1 and 234/2021BO2). Written informed consent was obtained from all participants and their legal guardians. Data collection took place between 10/2020 and 09/2022.

### Data collection

Video recordings, standardized according to the following procedure, were collected. Walking trials along a 6-m walkway were captured in the sagittal plane at a sampling rate of 50 Hz (AXIS P1375 network camera, 1920$\times$1080 pixels, 50 frames/s) [46]. The camera was mounted at a height of 50 cm and positioned at the center of the walkway, 2.5 m from the walking participants due to laboratory space constraints. To reduce distortions near the frame boundaries, only the central 4-m segment of each trial was used for analysis. Participants walked barefoot and wore tight clothing ending above the knees.

All participants were instructed to walk at a comfortable pace, turn at the end of the walkway, walk back, and repeat this procedure three times. After an initial test walk, two to three videos were recorded per participant, with each video capturing three passes along the walkway. Faces were anonymized in the recordings before further processing (DaVinciResolve19, Blackmagic Design).

### Human key-point estimation and event detection

#### Key-point estimation

Video-based motion analysis requires the estimation of the position and orientation (pose) of an object across all image sequences and the recognition of individual joints based on key-points. Before the key-points could be automatically extracted from the recorded sagittal-plane videos, a pre-trained Mask R-CNN model was used to infer a bounding box of the human in each frame of the video. A robust per-frame localization of the subject helps to (1) restrict subsequent processing to the human region, (2) reduce background clutter, and (3) improve the stability of keypoint detection. Mask R-CNN was selected because it is a well-established, instance-segmentation and detection framework that provides person localization and has been widely adopted as a pre-processing step for downstream human analysis tasks [47]. In the proposed pipeline, Mask R-CNN is used only for person localization (bounding box inference), not for pose estimation itself. Then the bounding box was taken to crop the image to the human region. As a next step, a pre-trained TCFormer model was applied on the cropped image to detect the key-points of the human. Zeng et al. provided the TCFormer model that they pre-trained on the COCO Whole Body dataset, which contains 133 key-points (17 for body, 6 for feet, 68 for face and 42 for hands) for each person [48]. The proposed approach subsequently utilized the TCFormer model to predict 12 points, representing the joint centers and anatomical regions of interest for 2D gait analysis (shoulders, hips, knees, ankles, heels, and toes). Only these 12 markers were selected and used for further calculations in the proposed approach. A linear smoothing procedure (see Sect. “Gait cycle computation”) was applied to accommodate individual prediction errors.

#### Joint angle computation

The key-points of the human body estimated in the previous step formed the basis for the calculation of the angles of the ankle, knee, and hip joints. To this end, the estimated key-points were used to define the joint centers of the lower extremities (hip, knee, ankle) and the associated lever arms (foot, lower and upper leg, and trunk). Joint angles are defined as the angles between the lines connecting the proximal and distal segments of each joint [49]. Only the key-points on the side facing the camera were used for the computation of the angle-trajectories, as these key-points were continuously visible and not occluded by the contralateral leg during swing. All computations were performed within the image plane, using the 2D pixel coordinates of the keypoints extracted from the sagittal video frames. For each frame, segment vectors were formed between adjacent joint centers (e.g., hip–knee and knee–ankle for the knee angle), and the joint angle was defined as the enclosed angle between the corresponding segment vectors in the sagittal image plane. All angles were expressed in degrees, representing anatomically consistent sagittal-plane joint rotations. The angle trajectories of the legs were captured alternately depending on the direction of walking. From these trajectories, the mean gait cycle time and three kinematic parameters for each participant were extracted: the minimum angle (Min), maximum angle (Max), and ROM of the joint angles throughout the gait cycle.

#### Event detection

A gait cycle is defined as the interval between two successive initial contacts of the same limb. It consists of the stance phase, initiated at foot strike, and the swing phase, which encompasses the forward progression of the leg until the subsequent contact [50]. To automate the identification of individual gait cycles in a given video, each video in the dataset was converted into a sequence of images. To identify gait events, a machine learning model was used to predict initial contact events. Specifically, a Gradient Boosting based classifier [51] was trained to predict the probability that a given frame is an initial contact event. The input features to the classifier were the angle estimates of the ankle, knee, and hip joints computed in the previous step. To train the classifier, experts manually labeled first-contact events in 40,519 frames from 32 videos from both cohorts (16 TD and 16 JIA). 20 randomly selected videos with a total of 25,732 frames constituted the training set, and the remaining 14,787 frames from 12 videos (6 TD and 6 JIA) were used for validation. Specifically, for each frame of each video, an expert labeled manually through visual identification whether it contained the event (i.e., heel strike) and the walking direction (left or right). The classifier, trained on the manually labeled frames, then predicted both the event type and walking direction for each frame. However, due to the similarity between adjacent frames, the classifier was prone to identifying multiple adjacent frames as events. To address this, two heuristic filtering rules were applied. First, within each sequence of consecutive frames classified as the same event, only a single middle frame was retained; if the sequence contained an even number of frames or only two frames, one of the central frames was randomly selected. Second, detections occurring within a threshold number of frames after the previous event were discarded, because true gait events cannot occur that closely together. The threshold was set to the average frame count between successive events in the training data, thereby filtering out likely false-positive detections. To evaluate the classifier, its predictions were tested on the 12 videos (14,787 frames) in the validation set.

#### Gait cycle computation

Based on estimated joint angles and predicted initial contact events, individual gait cycles were identified for all participants. To account for variability in cycle duration, all gait cycles were time-normalized to 100% of the gait cycle, and mean waveforms for ankle, knee, and hip joints were computed for each participant by averaging across all available cycles (on average 5.2 gait cycles per participant). Since key-point positions were estimated independently for each frame, a simple linear interpolation was applied to smooth the joint-angle trajectories prior to gait cycle computation.

### Validation

#### Validation against manually labeled ground truth

To establish the extent to which the proposed approach was able to accurately predict the location of the relevant key-points (i.e., the anatomical landmarks such as ankle, knee, and hip), the gait cycles generated by the proposed approach were compared with the gait cycles obtained from expert-labeled videos. To this end a random sample of two gait sequences from two different subjects with a total of 379 frames was labeled by an expert identifying 12 key-point locations per image as ground truth, which were then used as input to compute gait cycles. The performance of the key-point detection was evaluated by comparing these ground truth key-points and the predicted key-points from the proposed pipeline.

#### Validation against marker-based system

The proposed machine-learning-based approach for gait cycle computation from standard videos was compared with gait cycles derived from a marker-based optoelectronic approach. To validate the markerless approach against the 3D marker-based system, simultaneous recordings were performed on a subgroup of participants using both a standard video camera and a motion capture system. The presence of physical markers on the participants, necessary for the marker-based system, posed a potential challenge for the markerless approach, as they remained visible in the standard video recordings.

While videos with physical markers simplify the problem of joint location tracking, the goal of the validation was to demonstrate the accuracy of the markerless approach. Furthermore, to ensure an accurate comparison, it was essential to use the same set of gait cycles for both methods. Recording one walking trial with markers and another set without markers for the proposed approach could introduce variability in gait that makes a direct comparison problematic, in particular when working with young children. To address this, a novel validation technique was proposed that leverages advanced generative image models to remove physical markers from video recordings. Specifically, a deep learning–based image inpainting technique was employed to remove the physical markers [52]. By deriving gait cycles from the same video sequence, this method ensures direct temporal alignment, allowing for a more rigorous validation of the proposed markerless approach against the marker-based standard.

For the marker-based approach, a Vicon 3D Analysis with a ten-camera VICON FX motion capture system (sampling rate 120 Hz) was used. Additionally, three common cameras were set to record standardized videos in sagittal plane from each side and frontal plane independently from the Motion capture System. Participants were first asked to walk without markers at a self-selected speed on the 10-meter-long marked walkway of the gait laboratory to become familiar with the situation. Subsequently, 41 reflecting markers were attached to participants according to the Nexus Plug-in Gait model by a trained professional. The participants were again asked to walk at comfortable speed. This time the motion capture system as well as the cameras recorded the walk. Motion data from the marker-based system was further analyzed by Nexus Vicon Software.

**Fig. 2:**
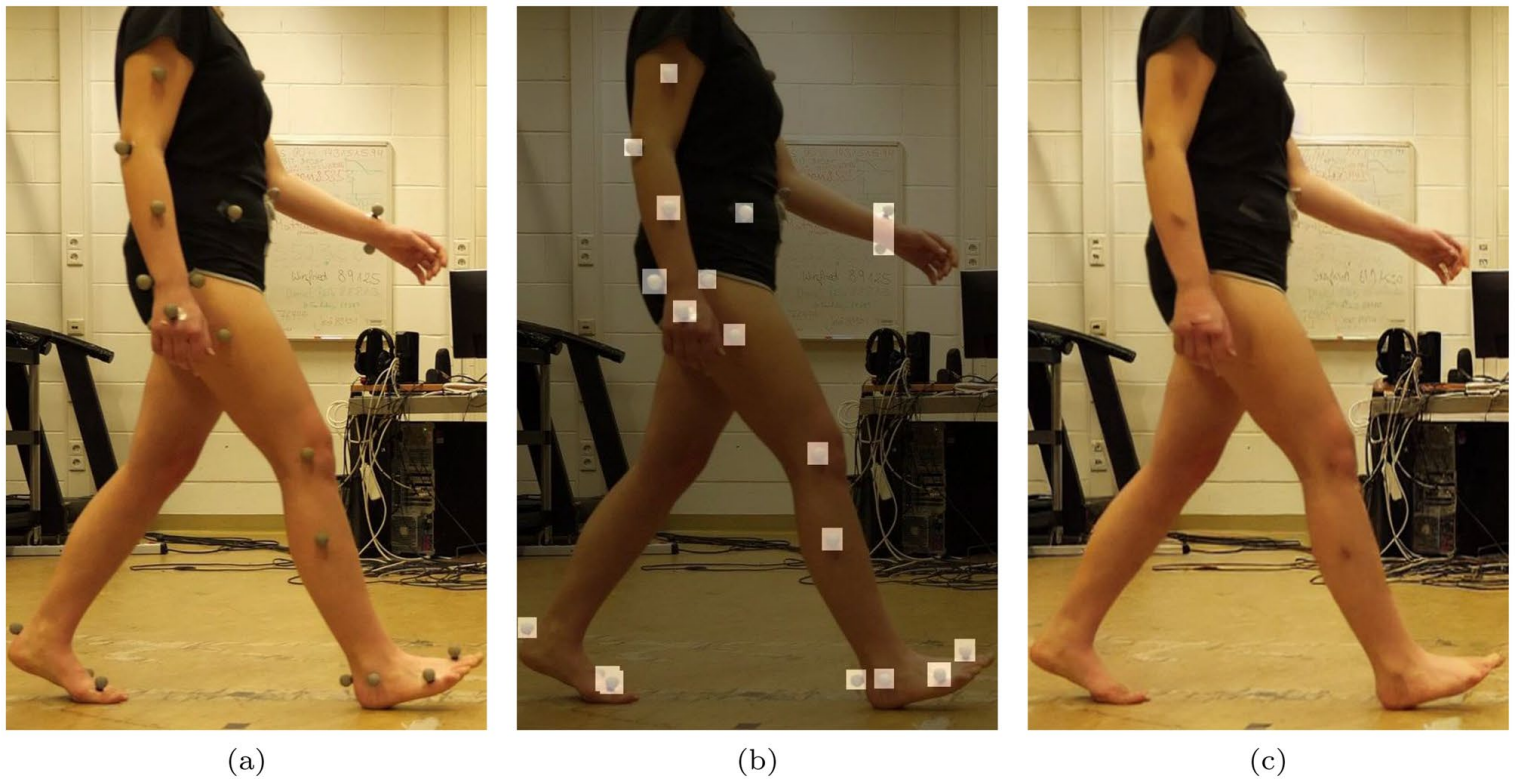
Illustrates marker detection and removal in the validation videos: (**a**) Raw frame, (**b**) Detected markers, (**c**) Inpainted frame used for markerless analysis

To utilize the videos that contained markers for the proposed markerless approach, the following steps were taken. First, the markers were detected from the regular videos using a deep learning-based object detector [53]. Next, a generative image inpainting technique [52] was employed to seamlessly remove the detected markers and fill in the missing regions with contextually appropriate visual data. This process ensures that the resulting markerless videos retain the original gait pattern, allowing for an accurate and direct comparison between the proposed method and the gait cycles derived from the marker-based optoelectronic motion capture system. Figure 2 illustrates the process of marker detection, removal, and inpainting with an example from the dataset.

The gait parameters were then extracted independently from both the markerless, video-based method and the marker-based motion capture system. To assess the concurrent validity of the proposed method, the degree of agreement between the two methods in capturing joint angle curves was determined.

### Statistical analyses

Consecutive patients with JIA and age-matched controls were evaluated. For demographics, means, medians, and ranges are reported.

All statistical analyses were conducted in Python. The primary analytical unit was the participant-level mean gait-cycle waveform for each joint (hip, ankle, and knee) and side, obtained by averaging all time-normalized gait cycles (0–100%) detected for that participant (see Sects. “Human key-point estimation and event detection”–“Gait cycle computation” for gait-cycle computation). Min, Max, and ROM were derived from each participant-level mean waveform. Mean gait cycle time was estimated for each subject.

#### Event detection performance

Initial-contact predictions were evaluated on the held-out validation set using a tolerance window of $\pm 4$ frames (0.08 s at 50 Hz). Accuracy, defined as the proportion of human-labeled initial-contact events for which a predicted event occurred within the tolerance window, is reported.

#### Validation of the proposed framework

The proposed framework underwent a two-stage validation process. First, kinematic accuracy was assessed at the waveform level against manual, frame-by-frame ground-truth labeling using a representative subset of two subjects. Subsequently, for the Vicon validation subgroup, the degree of agreement was quantified by comparing results against the 3D marker-based reference. To account for inherent temporal phase shifts and natural variations in walking speed, Dynamic Time Warping (DTW) was utilized to align the markerless waveforms with the marker-based reference sequences [54]. By optimally stretching or compressing the time axis, DTW ensures that comparison metrics, such as mean and difference waveforms, remain robust to variations in step duration and focus exclusively on morphological similarity.

Following DTW alignment, waveform similarity was quantified using Pearson correlation coefficients ($r$) computed between the marker-based and proposed-method gait-cycle waveforms on a per-participant basis (using scipy.stats.pearsonr). These correlations were summarized as the arithmetic mean of participant-level $r$ values for each joint.

To identify systematic method differences at specific phases of the gait cycle, a paired one-dimensional Statistical Parametric Mapping (SPM) $t$ test was performed on participant-level difference waveforms (marker-based minus proposed-method). Significant clusters over the 0–100% gait-cycle domain were interpreted as phases with consistent between-method deviations.

#### Group comparisons (JIA vs. TD)

Comparisons of gait outcomes between the control group, the overall JIA cohort, and the subgroup with active lower-limb arthritis were presented descriptively (tables of Min/Max/ROM and plots of mean trajectories with variability). Results were additionally shown stratified by predefined age bands (1.8–6.9, 7.0–12.9, 13.0–17.9 years). Statistical comparisons of joint kinematics between groups (JIA and TD) were performed using SPM.

## Results

### Participant characteristics

A total of 108 volunteers were video-recorded while walking, with one sagittal-plane video per participant included in this study. The JIA group comprised 60 patients, while the control group included 48 TD. The median age in the JIA group was 10.2 years (range: 1.8–16.1), and 10.3 years (range: 2.5–17.4) in the control group. Among participants with JIA, the majority were diagnosed with oligoarticular (46.6%) or polyarticular (38.3%) subtypes, while 6.8% were classified as having enthesitis-related JIA. The median disease duration was 49.0 months (range: 1–149 months). At the time of video acquisition, 13 JIA patients presented with active arthritis in at least one joint of the lower extremities (3 hip, 10 knee, and 5 ankle joints), with an average of 1.7 joints affected. Most of the patients were treated with biological or disease-modifying drugs (DMARD). To validate the proposed markerless method, a dedicated subgroup of six age-matched children per cohort (JIA and TD, 8–16 years) was compared against a marker-based reference standard. Younger children were excluded from this validation due to the anatomical constraints and technical difficulties associated with precise marker placement. Further group details are provided in Table  1.

**Table 1:** Demographic and clinical characteristics of patients with JIA and control groups of TD

	JIA Total	JIA Subgroup	TD Total	TD Subgroup
	($n = 60$)	($n = 6$)	($n = 48$)	($n = 6$)
Sex (Male:Female)	13:47	1:5	18:30	1:5
Median age (years, range)	10.2 (1.8–16.1)	13.0 (8.8–15.5)	10.3 (2.5–17.4)	13.5 (8.4–14.9)
Median height (cm, range)	141.0 (90.0–171.0)	158.3 (138.0–171.0)	148.3 (87.0–180.5)	163.0 (146.0–180.5)
Median weight (kg, range)	32.3 (12.0–74.5)	47.8 (30.0–69.0)	40.1 (13.2–95.4)	48.7 (36.0–69.6)
Median disease duration (months, range)	49.0 (1–149)	65.0 (4–149)	—	—
**JIA Subtypes**				
Oligoarticular arthritis (n,%)	28 (46.6)	2 (33.3)	—	—
Extended (n)	13	1	—	—
Persistent (n)	15	1	—	—
Polyarticular arthritis (n,%)	23 (38.3)	4 (66.7)	—	—
Systemic arthritis (n,%)	2 (3.3)	0 (0.0)	—	—
Psoriatic arthritis (n,%)	3 (5.0)	0 (0.0)	—	—
Enthesitis-related arthritis (n,%)	4 (6.8)	0 (0.0)	—	—
Unclassified arthritis (n,%)	0 (0.0)	0 (0.0)	—	—
**Joint Status**				
Patients with active arthritis (n)	13	1	—	—
Mean Joint Count (per patient with active arthritis)	1.7	1.0	—	—
**Median Physician GA (range)**	1.0 (0–6)	0.0 (0–3)	—	—
**Median Patient GA (range)**	1.0 (0–6)	0.5 (0–2)	—	—
**Median Pain Score (range)**	1.0 (0–7)	0.5 (0–3)	—	—
**Current Treatments**				
Biologicals (with MTX)	28 (15)	4 (2)	—	—
DMARDs monotherapy (MTX)	18 (15)	2 (2)	—	—
Corticosteroids with MTX	2	0	—	—
NSAIDs monotherapy	1	0	—	—
JAK-Inhibition	1	0	—	—
Untreated	10	0	—	—

Note: The columns JIA subgroup and TD subgroup represent age-matched subsets of the JIA total and TD total groups, respectively. For these subgroups, parallel recordings were conducted using both markerbased motion capture and a standard video camera, ensuring the necessary data for validating the proposed approach. JIA: Juvenile Idiopathic Arthritis, TD: typically developing children, Physician GA: Physician Global Assessment measured on a 0-10 Visual Analog Scale (VAS) with 0 = no activity and 10 = maximum activity, Patient Global Assessment assessed on a 0-10 VAS with 0 = best and 10 = worst, Pain Score: Self-reported Pain Score rated on a 0-10 Numeric Rating Scale (NRS) with 0 = no pain and 10 = maximum imaginable pain, MTX: Methotrexate, DMARD: disease-modifying antirheumatic drug, NSAID: non-steroidal anti-inflammatory drug, JAK: Janus-Kinase

### Key-point estimation and event detection

Following the steps outlined in Sect. “Human key-point estimation and event detection”, joint angles were computed from video images and key-points were estimated. For step segmentation, a Gradient Boosting based classifier was trained on 20 randomly selected videos, employing reference data manually annotated by experts for the key-points (initial contact event). Gait cycles were extracted from each video in the dataset based on key-points with an average of 16.3 and 15.5 complete cycles per participant for the right and left sides, respectively. These cycles were derived using an event-detection classifier that, when evaluated on the validation set of 12 videos, achieved an accuracy of 94.3% in predicting both heel strike events and walking direction.

### Validation

#### Validation against manually labeled ground truth

**Fig. 3:**
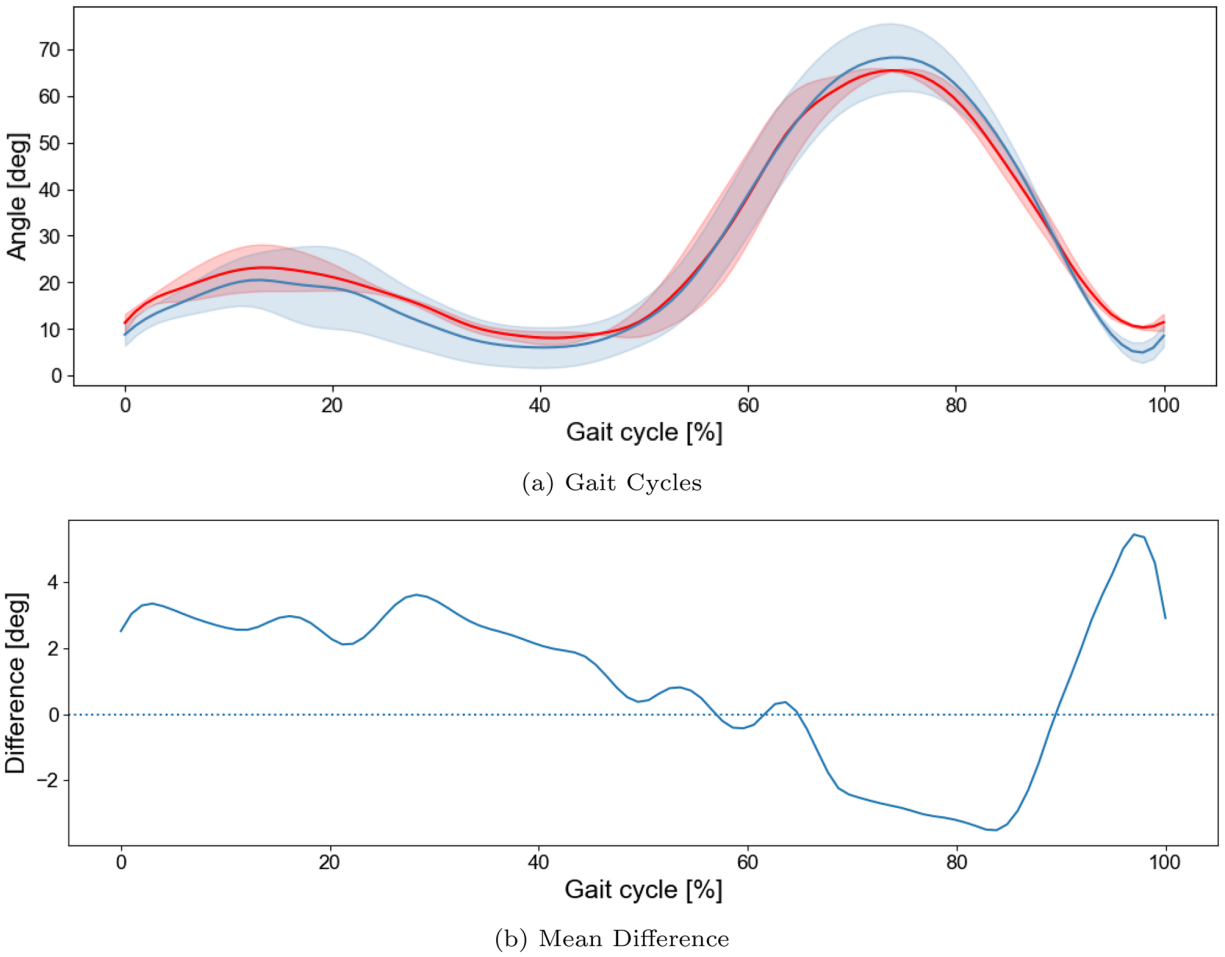
Validation of proposed method against manually labeled ground truth for knee flexion. *note:* (**a**) Comparison of gait cycles (knee flexion–extension) for Subject 42 and Subject 43 using manually labeled ground truth (red) and the proposed method (blue). (**b**) Mean difference waveform defined as manual minus proposed method, with the horizontal dotted line at zero. Deviations between methods were not statistically significant

To assess the accuracy of the proposed approach, knee gait-cycle waveforms were validated against a manual frame-by-frame ground truth. This initial comparison focused on the left knee of two representative participants (Subjects 42 and 43). Figure 3a shows that the knee flexion–extension trajectory estimated by the proposed method (blue) closely followed the trajectory computed from the manually labeled keypoints (red) across the gait cycle. Figure 3b presents the corresponding difference waveform (manually labeled minus proposed method), indicating small, phase-dependent deviations that remained within a few degrees throughout the majority of the gait cycle, with the largest differences occurring toward the end of the cycle. The one-dimensional SPM analysis did not identify any statistically significant differences between the two waveforms across the gait cycle. Because manual frame-by-frame annotation is labor-intensive, this evaluation was limited to two subjects. Agreement with the marker-based system was assessed in a larger validation sample in the next section.

#### Validation against marker-based system

**Fig. 4:**
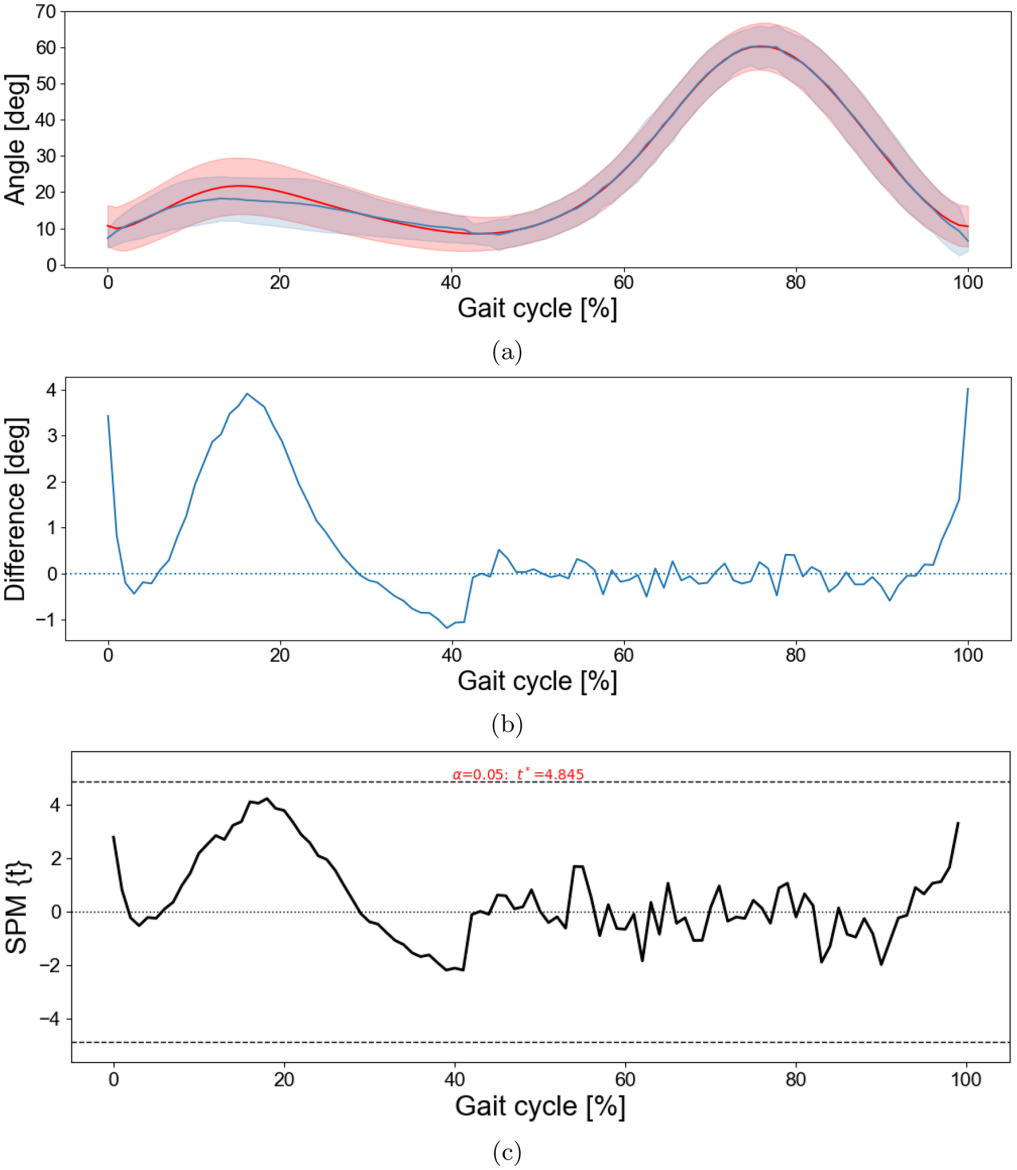
DTW-based comparison between the marker based approach and the proposed method for the knee joint. *note:* for each participant, the proposed-method gait cycle was aligned to the marker-based cycle using DTW. (**a**) DTW-aligned mean knee flexion–extension (marker-based: red; proposed: blue); shaded bands show $\pm 1$ SD across participants. (**b**) Mean difference (marker-based minus proposed) with zero line; shaded regions (if present) indicate SPM-significant intervals. (**c**) SPM$\{t\}$ from a paired one-sample $t$ test on subject-level difference waveforms; dashed lines show the critical threshold ($t^\ast$, $\alpha=0.05$). Positive clusters indicate larger knee angles in the marker-based approach

**Fig. 5:**
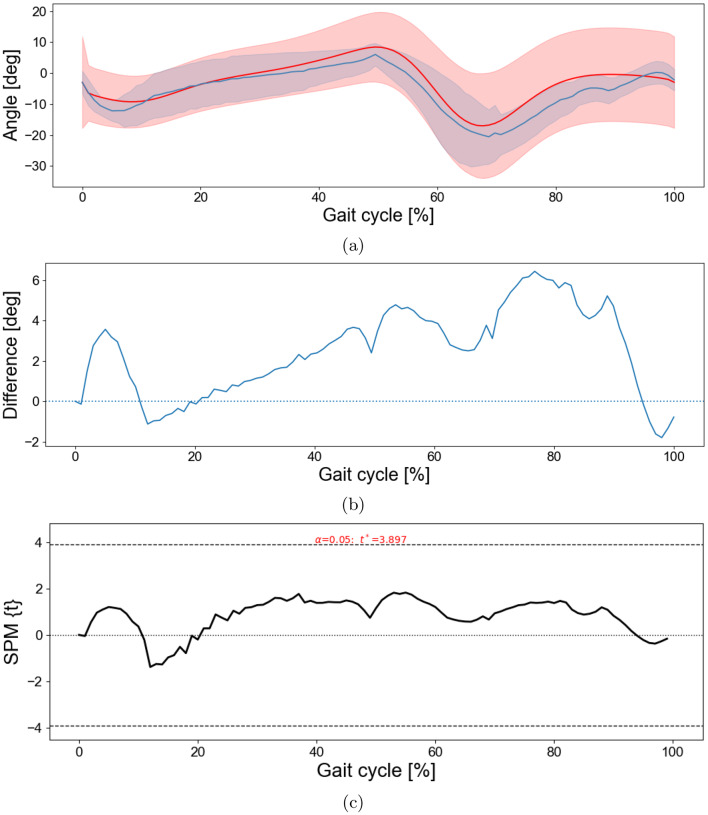
DTW-based comparison between the marker based approach and the proposed method for the ankle joint. *note:* for each participant, the proposed-method gait cycle was aligned to the marker-based cycle using DTW. (**a**) DTW-aligned mean ankle flexion–extension (marker-based: red; proposed: blue); shaded bands show $\pm 1$ SD across participants. (**b**) Mean difference (marker-based minus proposed) with zero line; shaded regions (if present) indicate SPM-significant intervals. (**c**) SPM$\{t\}$ from a paired one-sample $t$ test on subject-level difference waveforms; dashed lines show the critical threshold ($t^\ast$, $\alpha=0.05$). Positive clusters indicate larger ankle angles in the marker-based approach

**Fig. 6:**
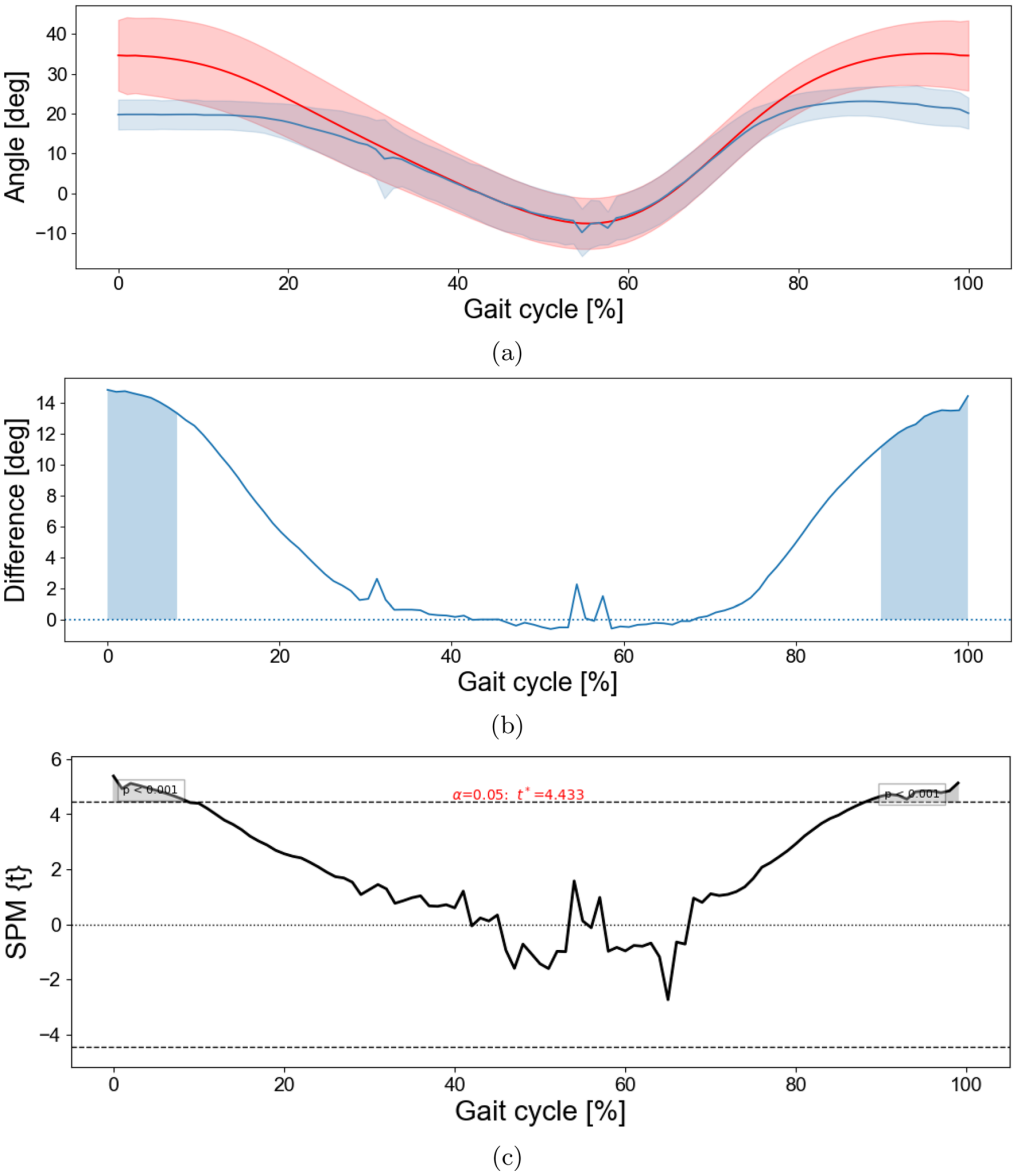
DTW-based comparison between the marker based approach and the proposed method for the hip joint. *note:* for each participant, the proposed-method gait cycle was aligned to the marker-based cycle using DTW. (**a**) DTW-aligned mean hip flexion–extension (marker-based: red; proposed: blue); shaded bands show $\pm 1$ SD across participants. (**b**) Mean difference (marker-based minus proposed) with zero line; shaded regions (if present) indicate SPM-significant intervals. (**c**) SPM$\{t\}$ from a paired one-sample $t$ test on subject-level difference waveforms; dashed lines show the critical threshold ($t^\ast$, $\alpha=0.05$). Positive clusters indicate larger hip angles in the marker-based approach

To assess agreement between the marker-based approach and the proposed method while accounting for potential temporal misalignment, a DTW-based analysis was applied to the joint angle trajectories. For each participant, this method aligned the proposed method’s gait cycle nonlinearly to the corresponding marker based cycle using DTW. Figure 4 shows the results for the knee joint. The DTW aligned mean curves showed very similar temporal patterns, with the proposed method slightly underestimating knee flexion during stance compared with the marker based approach (Fig. 4a). The difference waveform, defined as marker based minus proposed method, revealed modest positive deviations primarily during initial contact, in early stance and terminal swing, and smaller negative deviations during mid stance (Fig. 4b). A paired one-dimensional SPM $t$ test on the subject-level difference waveforms (Fig. 4c) identified no significant differences, with most of the differences being below 4 degrees. In the ankle joint, the paired one-dimensional SPM $t$ test of the difference waveforms at the subject level similarly showed no significant differences (Fig. 5). The only significant differences were detected in the hip joint during the first and last 10% (load response and terminal swing) of the gait cycle (Fig. 6).

Pearson correlation coefficients were calculated for each pair of gait cycles to compare the marker-based and proposed methods. To provide an overall measure of agreement across the 12 validation subjects, these individual values were averaged for each joint. This resulted in mean correlation coefficients of 0.95 for the knee, 0.95 for the hip, and 0.68 for the ankle joint.

Gait cycle time was used as an additional validation measure for the proposed markerless approach. Using the markerless method, mean gait cycle time was 1.00 s in the JIA subgroup (*n* = 6) and 1.02 s in the control subgroup (*n* = 6). For the same participants analyzed with the marker-based motion capture system, mean gait cycle time was 0.98 s (JIA) and 1.01 s (TD). Per-participant values for both methods are reported in Table 2. Overall, the mean difference between methods (proposed minus marker-based) was 0.009 s, and the paired t-test indicated no evidence of a difference from zero (t(11) = 1.42, *p* = 0.183; 95% CI: −0.005 to 0.023 s).

**Table 2:** Per-participant mean gait cycle time comparison between the marker-based approach and the proposed markerless method in seconds (**s**)

Participant	Marker-based (s)	Proposed (s)
**JIA**		
Subject 1	0.95	0.98
Subject 3	1.00	1.00
Subject 4	0.86	0.88
Subject 5	1.00	1.02
Subject 6	1.06	1.08
Subject 10	1.02	1.03
**TD**		
Subject 2	1.09	1.08
Subject 7	0.98	1.00
Subject 8	0.99	0.94
Subject 9	1.00	1.02
Subject 11	0.94	0.97
Subject 12	1.06	1.06

### Differences in gait characteristics between JIA and TD

**Fig. 7:**
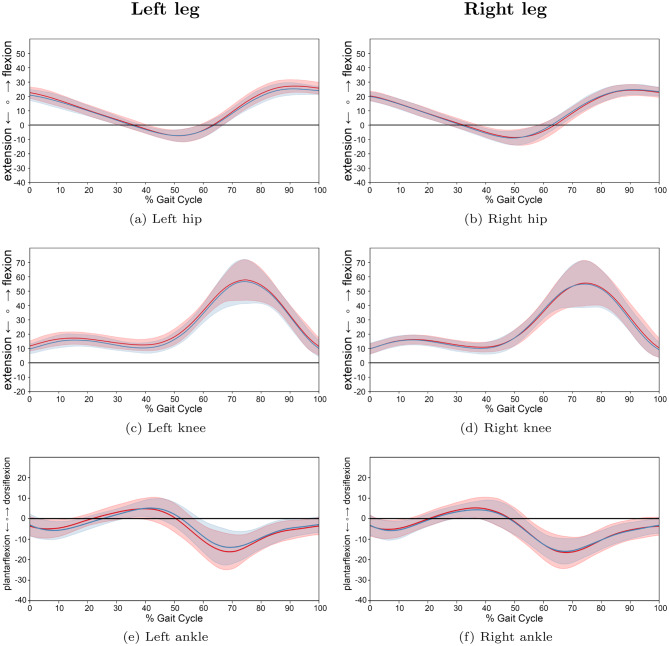
Comparison of gait cycles between patients with JIA and TD children for left and right legs. *note:* average angle trajectories (60 JIA patients: red; 48 TD: blue) of the hip, knee and ankle joint kinematics for both sides. Shaded areas indicate 95% confidence intervals. Data are normalized from heel strike to subsequent heel strike ($0$–$100$% gait cycle). SPM inference in appendix A, Figure A1

**Fig. 8:**
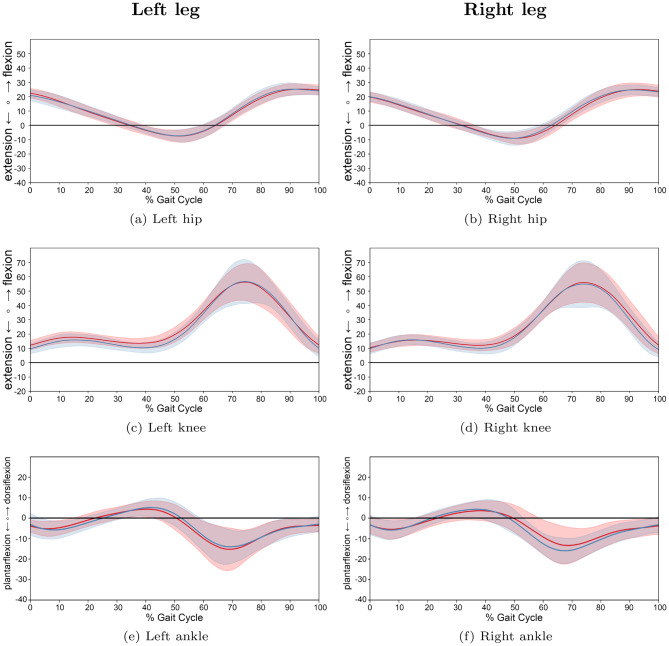
Comparison of gait cycles between patients with active JIA and TD children for left and right legs. *note:* average angle trajectories (13 JIA patients with active arthritis in the lower extremities: red line; 48 TD children: blue line) of the hip, knee and ankle joint kinematics for both sides. Shaded areas indicate 95% confidence intervals. Data are normalized from heel strike to subsequent heel strike ($0$–$100$% gait cycle)

**Fig. A1:**
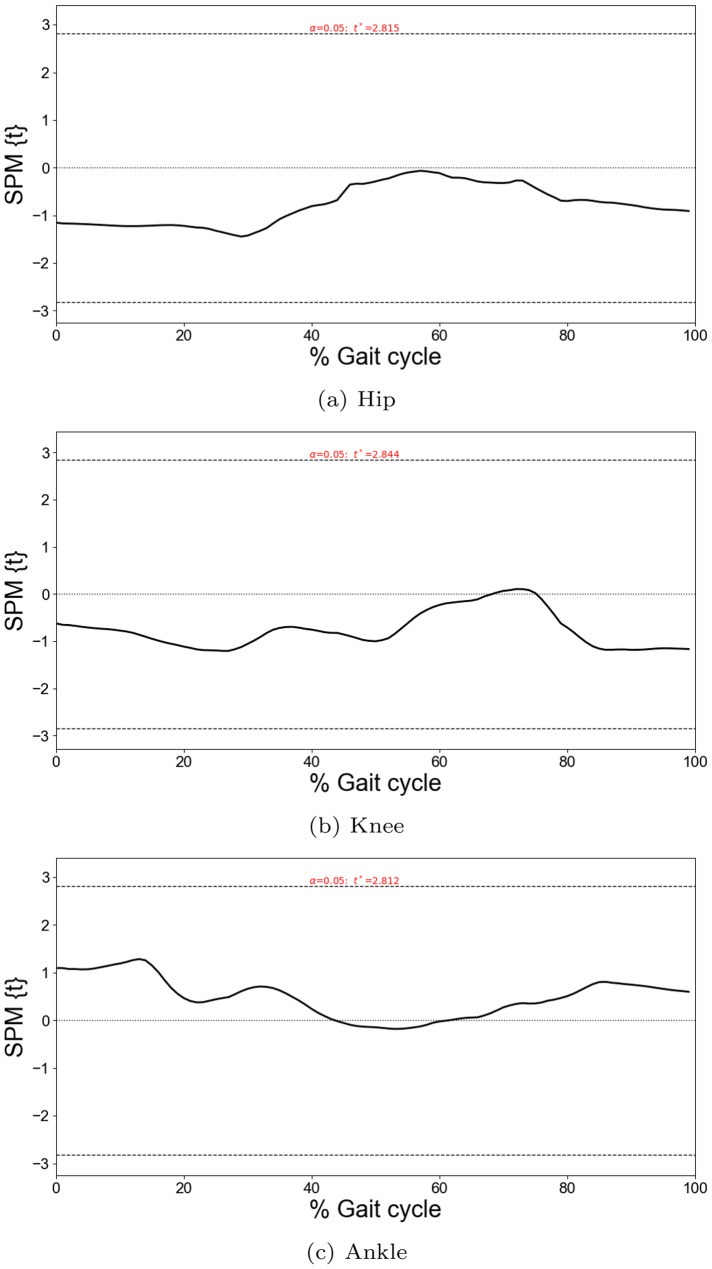
SPM results for joint kinematics comparing JIA and TD children (hip, knee, ankle)

Across all groups, comprising JIA, JIA with active arthritis of the lower limb, and TD, the sagittal-plane joint trajectories of the hip, knee, and ankle derived from the proposed markerless approach (Figs. 7 and 8) followed the expected physiological patterns. While no statistically significant differences were identified via SPM analysis between the JIA and TD groups (see SPM $t$ test plots in Figure A1), descriptive trends in the waveforms revealed group-specific deviations, particularly during key phases of the gait cycle.

The kinematic parameters are summarized in Table 3; here, smaller values represent greater extension or plantarflexion, while larger values indicate greater flexion or dorsiflexion. For the knee joint, the JIA group, and more notably the subgroup with active lower limb arthritis, exhibited a trend toward increased flexion during the first 60% of the gait cycle (stance phase) (Figs. 7 and 8). Specifically, the peak extension remained more flexed in the JIA and Arthritis group (left: $8.4^{\circ}$/ $8.8^{\circ}$ vs. $6.5^{\circ}$ right: $6.7^{\circ}$/ $8.3^{\circ}$ vs. $5.9^{\circ}$). Consequently, both maximum knee flexion and ROM were slightly reduced in this subgroup of patients with arthritis (Table 3). Regarding the hip joint, the values for maximum flexion and extension, as well as for ROM, showed only minor inter-group differences. In contrast, ankle joint kinematics in the active-arthritis group were characterized by reduced dorsiflexion and ROM (ROM: left: $18.6^{\circ}$ vs. $19.4^{\circ}$ right: $17.7^{\circ}$ vs. $20.3^{\circ}$) compared to TD controls (Figs. 7 , 8 and Table 3).

**Table 3:** Kinematic parameters in hip, knee, and ankle joints for patients with JIA, JIA with active arthritis, and control group of TD

Joint	**JIA** (*n* = 60)			**Arthritis** (*n* = 13)			**TD** (*n* = 48)		
	**Min**$^{\circ}$	**Max**$^{\circ}$	**ROM**$^{\circ}$	**Min**$^{\circ}$	**Max**$^{\circ}$	**ROM**$^{\circ}$	**Min**$^{\circ}$	**Max**$^{\circ}$	**ROM**$^{\circ}$
Left Hip	−7.5	27.0	34.5	−7.7	25.6	33.3	−7.4	25.4	32.8
Left Knee	8.4	57.4	49.0	8.8	54.9	46.1	6.5	55.9	49.4
Left Ankle	−15.9	5.5	21.4	−14.4	4.2	18.6	−14.1	5.3	19.4
Right Hip	−9.2	24.2	33.4	−9.1	24.8	33.9	−9.1	24.8	33.9
Right Knee	6.7	55.8	49.1	8.3	54.8	46.5	5.9	56.2	50.3
Right Ankle	−16.7	4.9	21.6	−13.4	4.3	17.7	−15.7	4.6	20.3

Note: Minimum (Min) values represent extension of hip or knee joint or plantarflexion of the ankle, and maximum (Max) values represent flexion of the hip or knee or dorsiflexion of the ankle joint. ROM: range of motion (calculated as the difference between maximum and minimum angles). All values are expressed in degrees of motion and are means

**Fig. 9:**
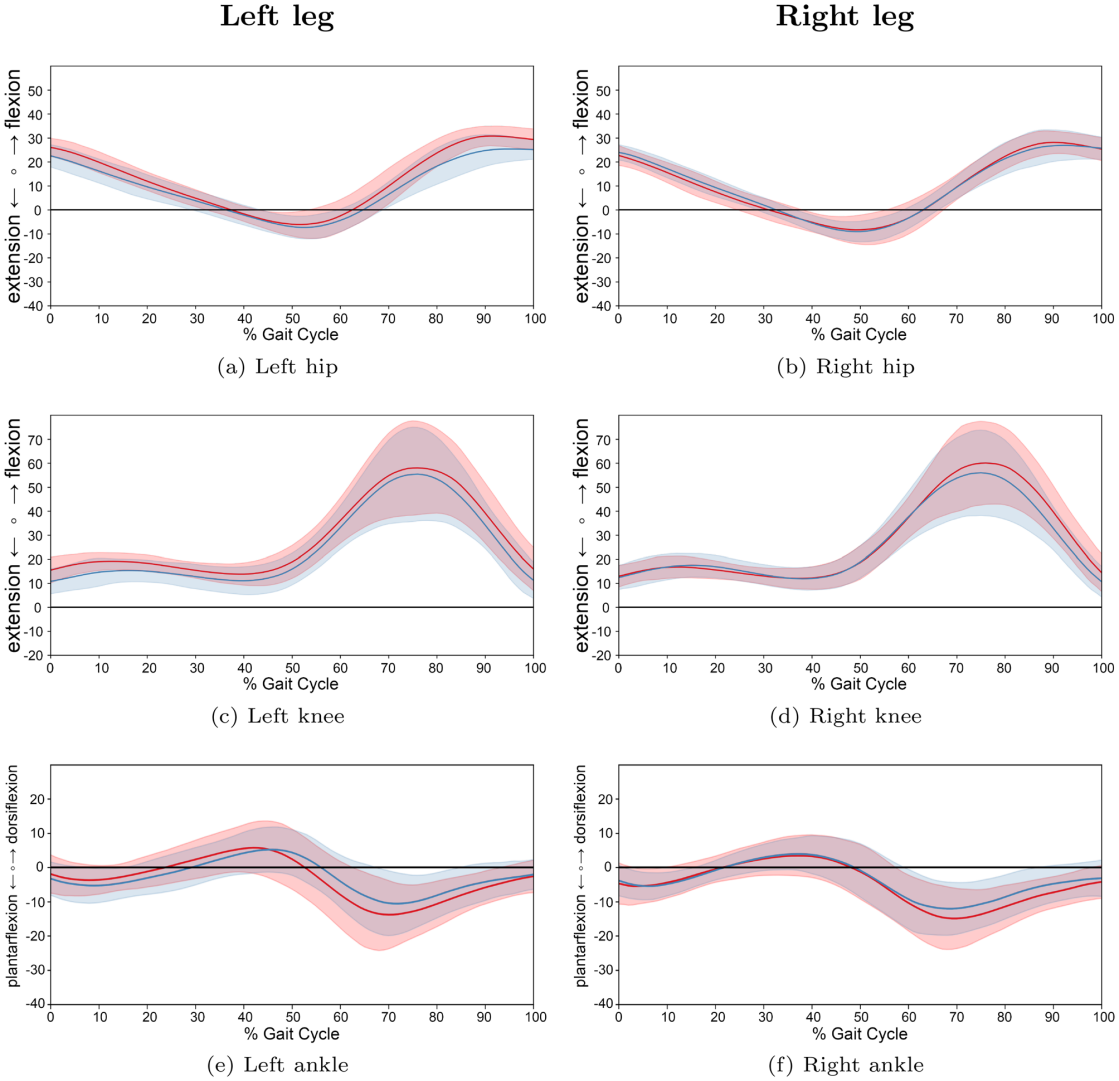
Comparison of gait cycles for subjects of age group 1. *note:* comparison between patients with JIA and TD children for left and right legs of age group 1 (preschool children and first graders (1.8 to 6.9 years)). Average angle trajectories (13 JIA patients: red line; 13 TD children: blue line) of the hip, knee and ankle joint kinematics for both sides. Shaded areas indicate 95% confidence intervals. Data are normalized from heel strike to subsequent heel strike ($0$–$100$% gait cycle)

**Fig. 10:**
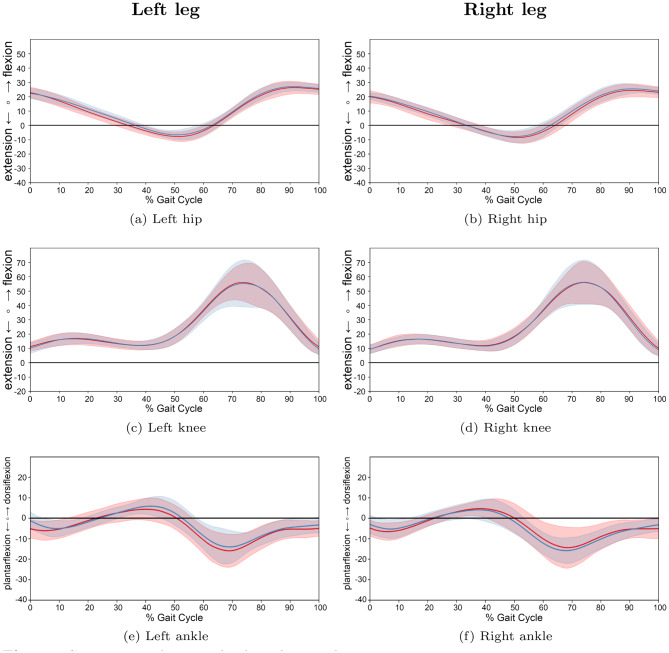
Comparison of gait cycles for subjects of age group 2. *note:* comparison between patients with JIA and TD children for left and right legs of age group 2 (school children (7.0 to 12.9 years)). Average angle trajectories (30 JIA patients: red line; 22 TD children: blue line) of the hip, knee and ankle joint kinematics for both sides. Shaded areas indicate 95% confidence intervals. Data are normalized from heel strike to subsequent heel strike ($0$–$100$% gait cycle)

**Fig. 11:**
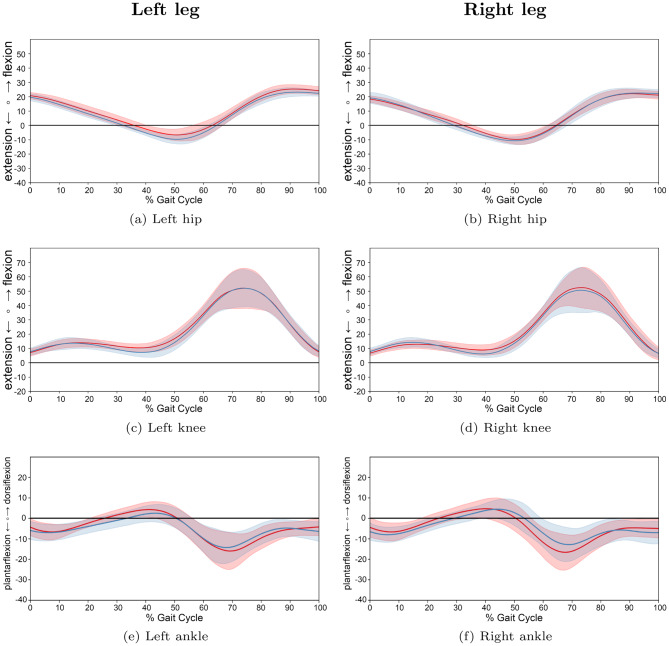
Comparison of gait cycles for subjects of age group 3. *note:* comparison between patients with JIA and TD children for left and right legs of age group 3 (adolescents (13.0 to 17.9 years)). Average angle trajectories (17 JIA patients: red line; 13 TD children: blue line) of the hip, knee and ankle joint kinematics for both sides. Shaded areas indicate 95% confidence intervals. Data are normalized from heel strike to subsequent heel strike ($0$–$100$% gait cycle)

For a more granular assessment, participants were stratified into three age-based cohorts: preschool and first grade children (Group 1: 1.8–6.9 years; 13 JIA, 13 TD), school-aged children (Group 2: 7.0–12.9 years; 30 JIA, 22 TD), and adolescents (Group 3: 13.0–17.9 years; 17 JIA, 13 TD). A descriptive analysis of the joint trajectories across these groups (Figs. 9, 10 and 11) revealed that the trends of increased knee flexion and ankle dorsiflexion in JIA were most prominent in the youngest and oldest cohorts. Furthermore, while movement variability was lower in the two older groups, the youngest cohort exhibited higher variability in their kinematic patterns.

## Discussion

This study presents an end-to-end workflow for the automated extraction of anatomical landmarks, gait events, and kinematic parameters from standard video recording. The framework provides reliable, clinically interpretable gait-cycle waveforms and demonstrates its practical utility through initial data from a JIA patient cohort.

While gait analysis is widely recognized as an effective diagnostic tool across various clinical populations, its implementation has traditionally required complex and costly hardware setups [41]. Recent research has sought to reduce these barriers by replacing physical markers and multi-camera systems with automated, video-based approaches using machine learning to detect anatomical landmarks. Several promising contributions have been made in this area [41]. This is, to the best of current evidence, the first study that (1) integrates systematic and automatic detection of initial contact events into a complete markerless gait analysis pipeline based solely on video data; (2) validates this pipeline on the exact same walking sequences used in marker-based motion capture, using a novel generative inpainting technique to remove the visual presence of markers; and (3) applies this pipeline to a pediatric sample including TD and patients with JIA aged 21 months and older, using a single-camera setup.

A critical component of video-based gait analysis is the accurate detection of gait events such as initial contact (heel strike). The proposed method automatically detects first-contact events by training a Gradient Boosting based classifier, achieving excellent accuracy of 94.3%. This suggests that a machine-learning-based automation of the detection of first-contact events is a feasible option. Traditional gait analysis relies on force plates to identify these events via ground reaction forces [35], requiring specialized hardware and limiting scalability. In the absence of force plates, gait events are typically identified manually, which is time-consuming and not scalable. Prior work has explored heuristic algorithms based on kinematic thresholds [55], as well as machine-learning-based methods trained on marker data [39, 56, 57] with comparable accuracy. However, these approaches have not been incorporated into end-to-end pipelines for fully video-based markerless gait analysis. The present study addresses this gap by training a dedicated classifier to detect gait events directly from joint-angle trajectories estimated from video data, by embedding this model into a completely markerless pipeline, and by demonstrating its practicality within the study population.

While marker-based systems are the standard reference for emerging technologies such as markerless approaches, the presence of markers introduces a significant methodological concern [58]: the visible reflective markers in the video may serve as unintended cues for the pose-estimation algorithm, potentially improving its performance in a way that does not generalize to marker-free settings. This issue has been discussed in the literature as a form of shortcut learning or domain shift [59]. Several prior studies have validated markerless systems on videos where markers were still visible [42, 56, 60]. In contrast, this study enables direct comparison with the marker-based reference on the same validation videos while avoiding visual leakage between methods by removing visible markers through generative inpainting.

The study findings demonstrate that a simple 2D markerless pipeline, utilizing a single sagittal-view camera, captures gait patterns in children and adolescents that closely resemble those obtained from a gold-standard 3D motion capture system. The alignment between the proposed method and a 3D motion capture system was supported by SPM analysis. While knee and ankle trajectories showed high consistency between systems, the hip joint exhibited transient statistical deviations during loading response and terminal swing. Although the offset in hip flexion of more than $10^{\circ}$ exceeds the $5^{\circ}$ clinical threshold [61], the strong waveform correlation ($r = 0.95$) suggests a systematic rather than random error. Consequently, while the systems are not interchangeable for absolute measurements, the markerless approach remains valid for clinical group comparisons (e.g., JIA vs. TD) where relative differences are the primary focus. This findings align with recent research [42, 62], confirming high proximal joint agreement ($r = 0.95$ for hip and knee joint) and lower ankle-parameter consistency ($r = 0.68$). Given the inherent gait variability in young children, who differ significantly from adolescents in velocity and direction [3], validation within the specific study population is essential [58]. The feasibility of this 2D approach extends recent research that applied OpenPose [63] to assess spatiotemporal parameters in toddlers [64]. By demonstrating that 2D markerless methods provide high morphological consistency despite this inherent variability, this study supports its feasibility and opens the door for more accessible gait analysis in pediatric clinical settings.

To date, few studies have examined kinematic gait parameters in children using markerless systems [65–67], and to the best of current knowledge, none have focused specifically on patients with JIA. A key contribution of this work is the demonstration that gait cycles can be reliably calculated using a markerless, video-based framework, thereby enabling the further assessment and monitoring of patients with JIA. The findings suggest that while JIA and TD children exhibit broadly similar kinematics, specific trends in knee and ankle trajectories emerge in cases of inflammatory joint activity. These indicate subtle but clinically relevant deviations in the subgroup with active arthritis. Although between-group differences in joint kinematics did not reach statistical significance, likely due to effective disease control in this well-treated cohort, the observed trends are clinically noteworthy and align with previous findings [17, 68]. Notably, the descriptive deviations were most prominent in the youngest cohort, those under 7 years of age. This represents an important finding, as marker-based gait analysis is often difficult to perform or, due to the need for cooperation and the physical placement of markers, is only feasible for children aged 6 years and older [38]. Consequently, this younger age group has remained largely under-researched in existing gait studies. Specifically, a tendency toward increased knee flexion was demonstrated in the JIA cohort at initial contact as well as during the swing phase. This reduced maximum knee extension at initial contact often reflects a pain-avoidance strategy, in which the full straightening of the joint is avoided during weight acceptance [17]. Furthermore, the descriptive results support a trend toward decreased dorsiflexion and ROM in the ankle in the active arthritis group, as reported by [69]. This is consistent with evidence that active arthritis restricts lower-extremity ROM, whereas low disease activity allows for improved or even normalized gait patterns [21]. Unlike previous studies that reported widespread reductions in ROM in the hip, knee and ankle joints [68–70], the present analysis found a slightly reduced ROM only in the knee and ankle joints, particularly in the subgroup with active arthritis. This discrepancy may be explained by walking speed and disease control; for instance, it was noted by [71] that ROM reductions in active JIA are often only detectable at faster velocities. Ultimately, the findings in this well-treated cohort indicate that nearly physiological gait patterns can be achieved by children with JIA during periods of disease remission.

From a clinical perspective, this markerless approach complements routine JIA assessments by providing objective longitudinal documentation of functional status. Although traditional joint examinations and patient-reported outcomes remain vital, repeated sagittal-plane kinematic measurements can help identify persistent, clinically relevant patterns, such as sustained knee flexion or reduced ankle range of motion, that may inform targeted interventions, including physiotherapy. The simplicity of this single-camera pipeline reduces hardware requirements and setup time compared to traditional marker-based systems, particularly beneficial for very young children and patients with active disease. Validated against a 3D marker-based reference, the resulting gait-cycle waveforms suggest that interpretable kinematic data can be derived from standard video. While not a replacement for full 3D analysis, this scalable 2D solution offers a practical tool for standardized gait monitoring in clinical settings where specialized laboratories are not available.

Some potential limitations should be considered when interpreting the findings. First, while this single-center study involves a limited sample size, it represents one of the largest JIA gait analysis cohorts published to date. The distribution of JIA subtypes within the overall study cohort is broadly representative of the typical clinical spectrum observed in larger pediatric rheumatology cohorts [11]. Therefore, there is little reason to assume that these findings would differ significantly across other centers. Nevertheless, the validation subgroup (6 JIA patients) did not include any cases of enthesitis-related JIA. Since this clinical phenotype is frequently associated with pronounced lower-extremity involvement, its absence in the subgroup analysis may affect the generalization to the broader JIA population.

Second, the markerless method used in this study captures data from only one side of the body per walking direction. While this limits the simultaneous analysis of both sides, it improves accuracy by focusing only on the anatomical landmarks that are clearly visible and not occluded by the contralateral limb. Moreover, the computation of joint angles remains transparent, as the detected landmarks are displayed frame by frame. Third, videos were recorded at 50 Hz (vs. 120 Hz for the marker-based approach). Although time-normalization and waveform-based comparisons reduce sensitivity to sampling-rate differences, the lower video frame rate may still limit the precision of event timing and peak estimates, and future work should assess this via sensitivity analyses. Fourth, although this method lacks the anatomical detail of 3D motion capture [34], it demonstrates high waveform consistency with gold-standard measures. Consistent with prior research [42], agreement was very high for knee and hip trajectories, though lower at the ankle. While a transient hip flexion offset exceeds the 5 $^{\circ}$ clinical threshold [61], the strong morphological agreement confirms that the systems, though not interchangeable for absolute measurements, are valid for clinical group comparisons (e.g., JIA vs. TD).

Finally, the challenges associated with marker-based detection in early childhood limited direct validation against the gold standard to children aged 8 and older. Although a comparison for the youngest cohort is still pending, this gap underscores the clinical need for markerless alternatives: It is precisely the difficulty of performing conventional 3D analysis in this age group that makes the development of accessible, markerless tools essential.

Future research should focus on utilizing multi-camera fusion and age-specific training datasets to refine anatomical accuracy in younger cohorts. Furthermore, multi-center studies could broaden the understanding of gait alterations across various musculoskeletal and neurological conditions beyond JIA. To facilitate routine adoption, further work should focus on integrating pose estimation models into efficient, user-friendly workflows that allow for seamless in-clinic data processing. Ultimately, bridging these technical and practical gaps will establish markerless analysis as a robust, universal standard for objective gait assessment in pediatric clinical care.

## Conclusions

This study shows that video-based gait analysis can provide reliable and valid gait data even in young children. Differences in gait patterns between children and adolescents with and without JIA were generally small, likely due to effective disease control. Compared to traditional methods of gait assessment, markerless video-based gait analysis offers several advantages: it imposes fewer logistical barriers and can be applied in a variety of settings, including outpatient clinics and even at home. Due to its simplicity, the proposed markerless approach is especially suitable for repeated measurements over the course of disease and may help to improve understanding of functional gait abnormalities in JIA. In conclusion, single-camera, markerless, video-based approaches can provide clinicians with a practical and sufficiently accurate tool for gait assessment across all pediatric age groups.

## Data Availability

Availability of data and materials. The datasets supporting the conclusions of this article are included within the article and its additional files. Raw video data from patients and participants are not publicly available to preserve individuals’ privacy under the European General Data Protection Regulation. Data are, however, available from the authors upon reasonable request and only after permission of the Ethics Committee at the Medical Faculty and the University Hospital Tuebingen. Code will be freely available under https://github.com/aseembehl/markerless-gait-analysis-jia-ml-pipeline.

## List of abbreviations

2D: Two-dimensional
3D: Three-dimensional
CNN: Convolutional Neural Network
DMARD: Disease-Modifying Antirheumatic Drug
DTW: Dynamic Time Warping
GA: Global Assessment
ILAR: International League of Associations for Rheumatology
JAK: Janus Kinase
JIA: Juvenile Idiopathic Arthritis
Max: Maximum
Min: Minimum
MTX: Methotrexate
NRS: Numerical Rating Scale
NSAID: Non-Steroidal Anti-Inflammatory Drug
ROM: Range of Motion
SPM: Statistical Parametric Mapping
TD: Typically developing participants
TCFormer: Token Clustering Transformer
VAS: Visual Analog Scale
